# Development and evaluation of a core genome multilocus sequence typing scheme for *Paenibacillus larvae*, the deadly American foulbrood pathogen of honeybees

**DOI:** 10.1111/1462-2920.15442

**Published:** 2021-03-02

**Authors:** Alicia C. Bertolotti, Eva Forsgren, Marc O. Schäfer, EuroPLarva Consortium, Fabrice Sircoulomb, Nicolas Gaïani, Magali Ribière‐Chabert, Laurianne Paris, Pierrick Lucas, Claire de Boisséson, Joakim Skarin, Marie‐Pierre Rivière

**Affiliations:** ^1^ Anses, Sophia‐Antipolis Laboratory Unit of Honey Bee Pathology Sophia Antipolis France; ^2^ Department of Ecology Swedish University of Agricultural Sciences Uppsala Sweden; ^3^ Federal Research Institute for Animal Health Friedrich‐Loeffler‐Institut Greifswald Insel Riems Germany; ^4^ Anses, Ploufragan‐Plouzané‐Niort Laboratory Unit of Viral Genetics and Biosafety Ploufragan France; ^5^ Department of Microbiology National Veterinary Institute Uppsala Sweden

## Abstract

*Paenibacillus larvae* is the causative agent of the fatal American foulbrood disease in honeybees (*Apis mellifera*). Strain identification is vital for preventing the spread of the disease. To date, the most accessible and robust scheme to identify strains is the multilocus sequence typing (MLST) method. However, this approach has limited resolution, especially for epidemiological studies. As the cost of whole‐genome sequencing has decreased and as it becomes increasingly available to most laboratories, an extended MLST based on the core genome (cgMLST) presents a valuable tool for high‐resolution investigations. In this study, we present a standardized, robust cgMLST scheme for *P*. *larvae* typing using whole‐genome sequencing. A total of 333 genomes were used to identify, validate and evaluate 2419 core genes. The cgMLST allowed fine‐scale differentiation between samples that had the same profile using traditional MLST and allowed for the characterization of strains impossible by MLST. The scheme was successfully used to trace a localized Swedish outbreak, where a cluster of 38 isolates was linked to a country‐wide beekeeping operation. cgMLST greatly enhances the power of a traditional typing scheme, while preserving the same stability and standardization for sharing results and methods across different laboratories.

## Introduction

Honeybees (*Apis mellifera*) are the major pollinator for crops that depend on animal pollination (~35% of the global food production) (Potts *et al*., 2010). They are therefore a key species for food security, a healthy economy and sustainable agriculture (Klein *et al*., 2007). They also contribute significantly to maintaining biodiversity by pollinating wild flowers (Hung *et al*., 2018). Due to their important role, dramatic colony losses and population declines have attracted attention. The sources of these mass deaths are multiple, with pesticides, climate change and, in particular, pathogens playing a major part (Winfree *et al*., 2009; Genersch, 2010; Potts *et al*., 2010; Cresswell *et al*., 2012; Henry *et al*., 2012; Vanbergen and Initiative, 2013; Woodcock *et al*., 2017).

Of these pathogens, American foulbrood (AFB) is the most deleterious infectious disease affecting honeybee brood. The disease is caused by spores of the bacteria *Paenibacillus larvae*. AFB is a notifiable disease in the EU (Council Directive 92/65/EEC 1992) and is registered on the list of the World Organization for Animal Health (OIE 2020). The symptoms of the disease are characterized by a mosaic brood pattern, a brownish semi‐fluid, glue‐like consistency of the affected larvae and a characteristic foul odour of the infected frames (OIE 2016).

The massive production of extremely long‐lived bacterial endospores in diseased colonies makes the control of AFB difficult (Dobbelaere *et al*., 2001b). Burning the symptomatic diseased colonies (including bees, brood comb and hive equipment) is widely considered the only workable control method and is the usual legal requirement in most European countries. Current legislation does not allow European beekeepers to use antibiotics to control AFB (Commission regulation (EU) 37/2010). Their use, such as is the practice in several countries outside EU, is unsustainable since it does not eliminate the bacterial spores that drive the epidemiology but instead masks the symptoms of the disease. Furthermore, the overuse of antibiotics may lead to resistant strain emergence (Evans, 2003; Alippi *et al*., 2007; Krongdang *et al*., 2017).

Five different *P*. *larvae* genotypes have been described so far, being classified based on the amplification of enterobacterial repetitive intergenic consensus (ERIC) sequences: ERIC I to V (Genersch *et al*., 2006; Beims *et al*., 2020). Numerous epidemiological studies show that ERIC I and II are the genotypes predominantly found in colony foulbrood outbreaks (Loncaric *et al*., 2009; Morrissey *et al*., 2015), and therefore the most important to focus on in connection with honeybee health. These two genotypes differ mainly by their virulence: ERIC I is the less virulent type on individual level killing larvae in, on average, 12 days compared with 7 days for the ERIC II type (Genersch *et al*., 2005; Genersch *et al*., 2006; Rauch *et al*., 2009).

Besides ERIC genotyping, an MLST scheme based on seven housekeeping genes has been described (Morrissey *et al*., 2015). This scheme allows for a better understanding of *P*. *larvae* population structure, particularly in its native range. It has proven useful to determine distribution and biogeography of the species and limited epidemiological success for tracking a small outbreak of AFB in Jersey (Morrissey *et al*., 2015).

With the advances of sequencing technologies, particularly the substantial decrease in costs of sequencing whole genomes (WGS), there is now an opportunity to extend the traditional seven gene MLSTs to thousands of genes, using either the whole genome (wgMLST) or the core genome (cgMLST), where genes present in all isolates are compared. These schemes offer a higher discriminatory power and are routinely used in bacterial epidemiological and diagnostical studies of both human and animal pathogens (de Been *et al*., 2015, de Been *et al*., 2015a; Ruppitsch *et al*., 2015; Ghanem and El‐Gazzar, 2018; Pearce *et al*., 2018; Sankarasubramanian *et al*., 2019). A small study has already demonstrated the potential resolution of cgMLST in *P*. *larvae* by tracking a localized outbreak in Sweden (Ågren *et al*., 2017). Despite these efforts, no standardized scheme for typing *P*. *larvae* using WGS is currently available. For this reason, we have developed a standardized cgMLST created with 199 *P*. *larvae* genomes that represent the highest possible geographical and temporal diversity. We used this scheme to identify possible outbreak clusters using an additional 134 genomes from several regions in Sweden, demonstrating the power of our scheme even when dealing with closely related isolates.

## Results

### Genome sequencing and assembly

In total, 333 genomes were used in this study. Assembly sizes ranged from 3 645 620 to 4 557 603 bp (mean = 4 111 965; SE = 12 040). Number of contigs varied from 250 to 1695 (mean = 579; SE = 11). The GC content varied from 43.3% to 45.1% (mean = 44.10; SE = 0.01). As expected from their genotypes, ERIC I genomes had, on average, 465 341 bp more than ERIC II genomes with ERIC I genomes being approximately 4 200 000 bp in size.

### MLST sequence typing

MLST‐typing was used to identify and check proper clustering using the stable cgMLST scheme. A total of 12 different STs were identified, 10 belonging to the ERIC I genotype and two belonging to the ERIC II genotype (Supplementary Table 1).

Eight clones (2.4% of samples) could not be attributed to STs using the 7‐loci MLST due to one or two loci not being found in their genome (Supplementary Table 2). The stable cgMLST was used to infer likely STs for these samples (Fig. 3).

### cgMLST development

In total, 685 targets were discarded. Of these, 87 were discarded due to the start codon not being at the beginning of the gene, 272 had more than one single stop codon at end of the gene, 347 had homologous genes with BLAST overlap ≥100 bp and identity ≥90.0%, 218 overlapped with other genes >4 bases, nine had BLAST hit with overlap ≥100 bp and identity ≥90.0% in excluding sequences.

The 148 genomes used to validate the stability of the cgMLST scheme all contained >95% of the targets, with a mean of 98.04% (SE = 0.2). Alleles in each locus varied from 26 to 1 (mean = 4.58; SE = 0.06) (Supplementary Fig. 1). The cgMLST can be found in https://www.cgmlst.org/ncs .

The final total number of core genome targets identified was 2419 loci (2 126 941 bp), representing 49.6% of the seed genome. The number of accessory targets identified was 1182 loci (891 873 bp) representing 20.9% of the genome.

### cgMLST evaluation and outbreak analysis

Building a stable and reliable cgMLST useful for tracking localized outbreaks of very similar samples, while being broad enough to encompass the entirety of the species genetic variability, is a tricky balance. In order to evaluate the resolution of this stable cgMLST, 24 samples belonging to an outbreak in 2014 in Gotland, Sweden, were analysed and compared with results published using two separate *ad hoc* cgMLST schemes, one per ERIC genotype (ERIC I and ERIC II). This comparison was used to evaluate the resolution of the stable cgMLST combining the two ERIC types. It represents an ideal dataset as (i) it has already been characterized and therefore differences can easily be quantified, (ii) it is a local outbreak of closely related samples and (iii) it includes both genotypes, ERIC I and II.

Using the stable cgMLST scheme, the highest number of allelic differences identified were the distance between genotypes ERIC I and ERIC II, where isolates were separated by 1198 alleles. Within ERIC II, two clusters separated by 18 alleles could be observed. Within ERIC I there were two clusters, representing two different STs, which were separated by 670 alleles. However, there were very few allelic differences (min = 0, max = 3, average = 1) within the clusters of isolates.

**Fig 1:**
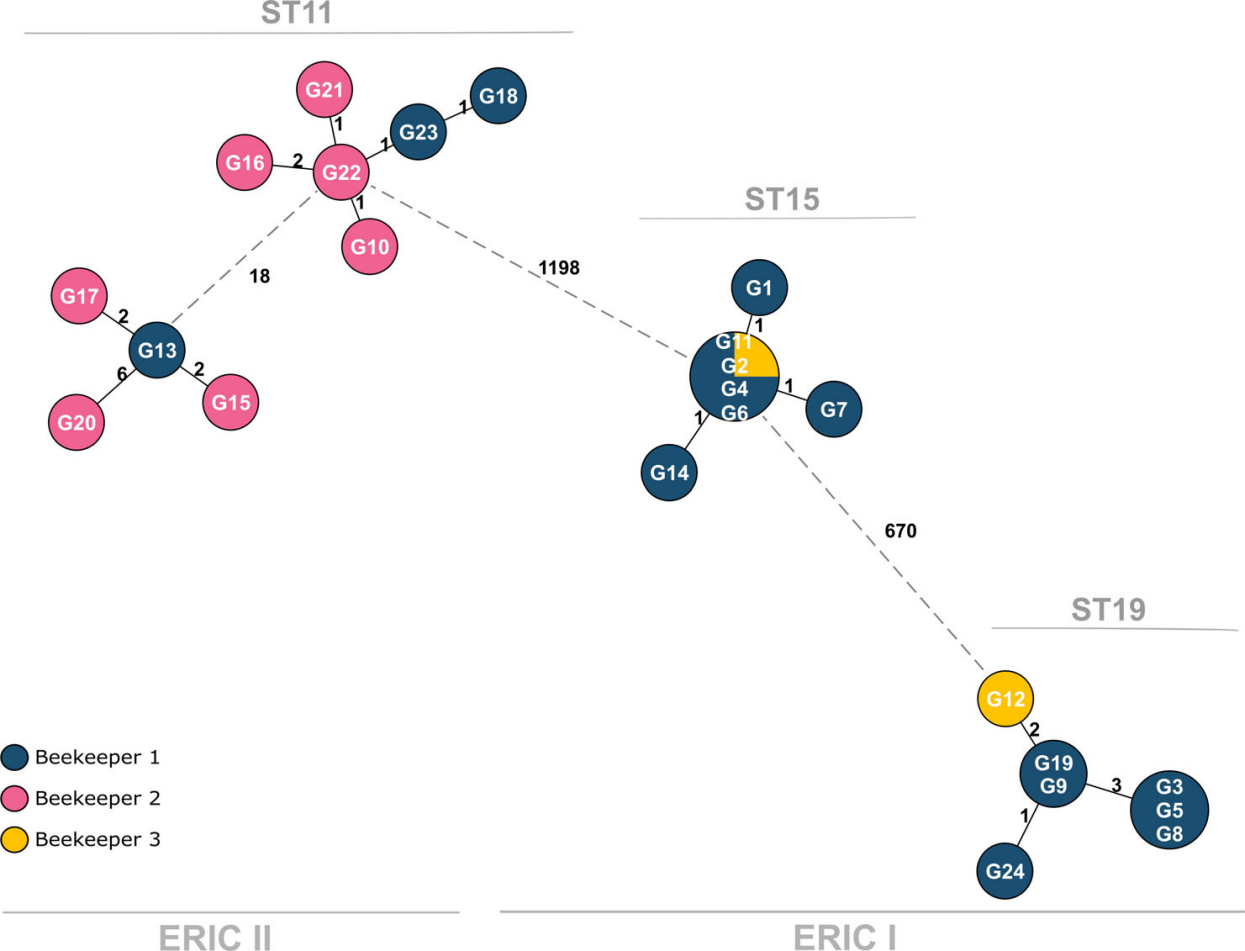
Cluster analysis of 24 isolates based on allelic differences using the stable core genome MLST. A minimum spanning tree showing number of allelic differences between isolates of the AFB outbreak on the island of Gotland, Sweden, in 2014. Results are based on 2419 target genes. ST11, ST15 and ST19 represent the seven‐gene MLST types of the clusters. Dotted lines are used here to indicate the four different apparent clusters.

The differences within the clusters strongly suggest that the isolates are very closely related and likely to be four distinct outbreak clones as observed by Ågren *et al*. (2017). When considering isolate origin, findings of this study concur with those of the study published in 2017 showing that beekeeper number one was responsible for transmitting all four outbreak clones of AFB to the other two beekeepers (Fig. 1).

**Fig 2:**
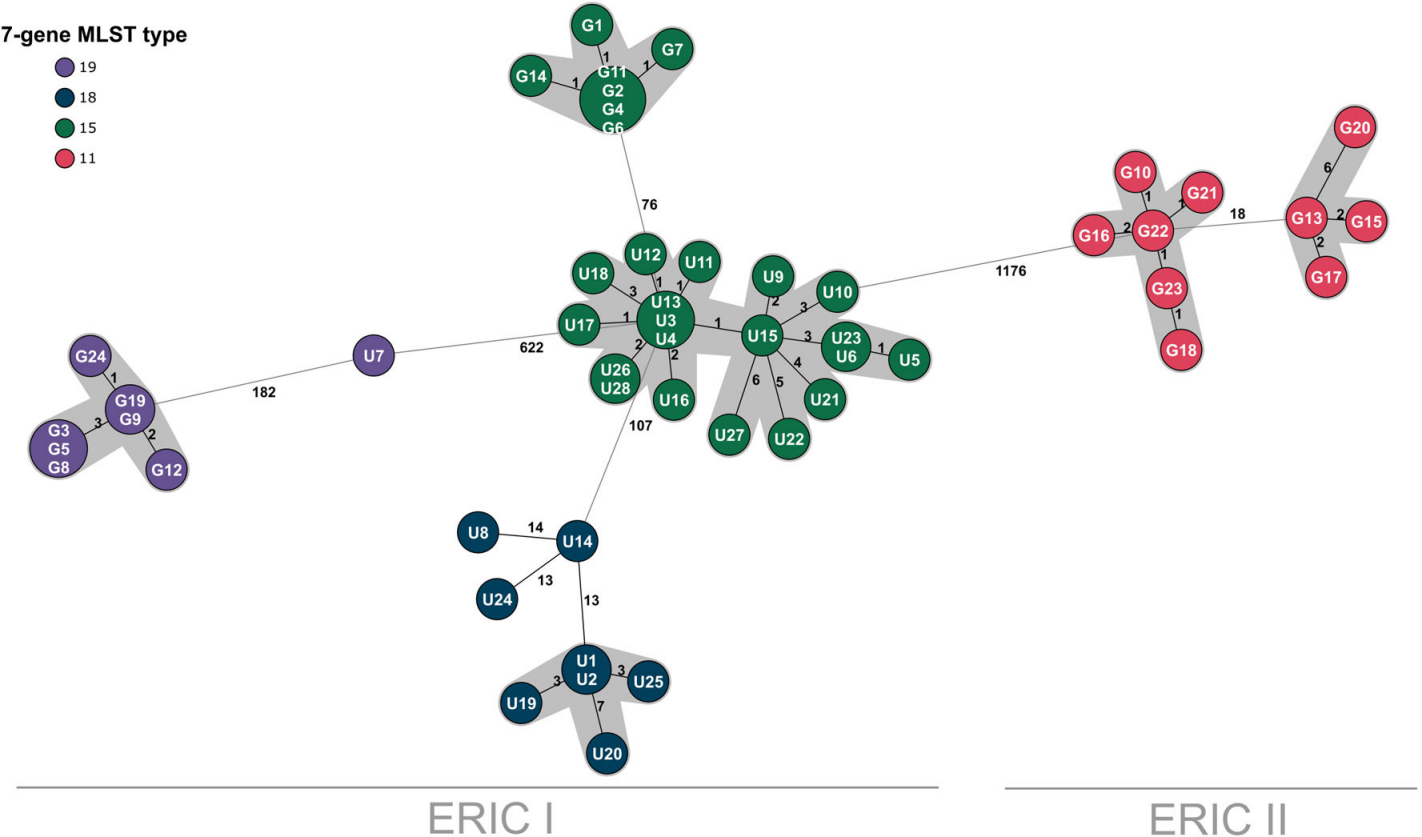
Cluster analysis of 52 isolates based on allelic differences using the stable core genome MLST. A minimum spanning tree showing number of allelic differences between isolates of the AFB outbreak on the island of Gotland and the unrelated outbreak in Uppland County, Sweden, in 2014. Results are based on 2419 target genes. Closely related clusters (<10 allelic differences) are highlighted in grey. Isolates starting with ‘G’ are from Gotland and ‘U’ are from Uppland.

An additional 28 isolates from apiaries from mainland Sweden (Uppland county) were added to further evaluate the sensitivity of the scheme (Fig. 2). These isolates were not linked to the Gotland outbreak. Results show that some isolates, although being of the same ST as the Gotland samples, can easily be distinguished from the outbreak event. For instance, U7 is 182 alleles apart from the Gotland samples of the same ST. The same conclusions are true for samples of ST 15, where Uppland samples were 76 alleles apart from the samples of Gotland origin. These fine‐scale results could not have been observed using a traditional MLST scheme.

Finally, the scheme was evaluated for its applicability on a dataset of 134 bacterial genomes isolated from symptomatic brood in Sweden during 2016–2019.

**Fig 3:**
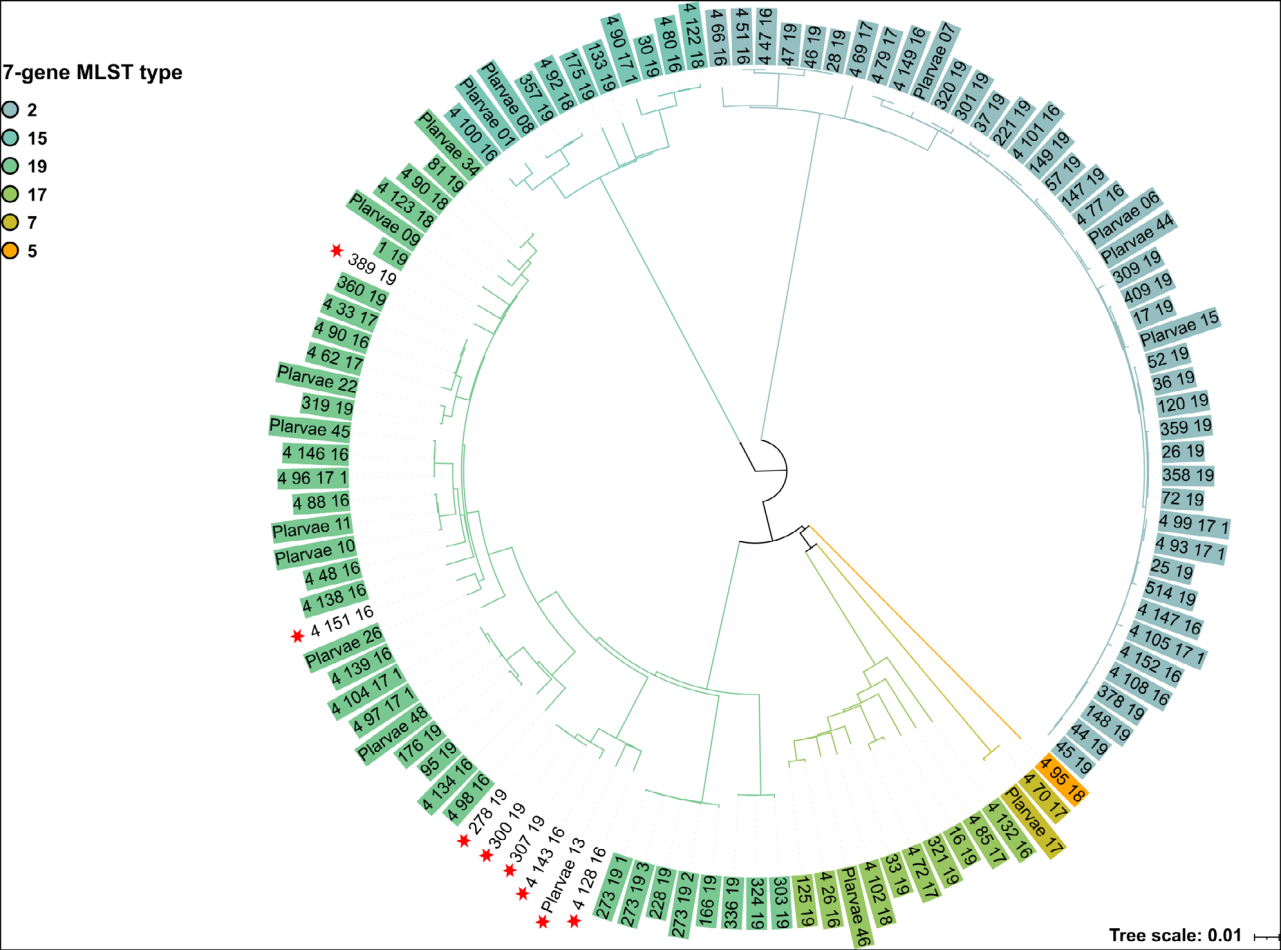
Neighbour‐joining tree of the 113 ERIC I SNRL isolates from Sweden. Groups very clearly formed by ST. Red stars highlight the samples that were not possible to identify using the seven‐gene MLST typing scheme due to missing loci. Here it is very clear that the eight samples belong to ST19. ERIC II isolates are not represented here as all STs could be determined using seven‐gene MLST.

The stable cgMLST was initially used to type eight isolates that were unsuccessfully typed using the seven‐gene MLST loci (Fig. 3). This failed typing was due to one or several loci of the scheme not being found in the genomes (Supplementary Table 2).

**Fig 4:**
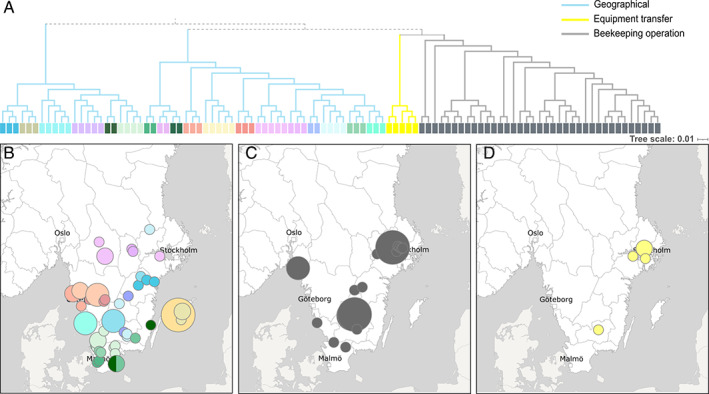
(A). A neighbour‐joining tree based on cgMLST loci, showing the 105 samples that group into 21 clusters separated by <10 alleles. Maps of Sweden showing: (B) the 19 clusters that group by geographical location, (C) one cluster of 38 closely related genomes which can be linked to a beekeeping operation active in southern and central Sweden and (D) five bacterial isolates from three counties likely associated with transfer and sales of beekeeping equipment and bees.

Furthermore, the samples were processed using Ridom SeqSphere+ using default settings to identify possible outbreak clusters and to determine relatedness between the genomes. Using the allele difference threshold of 10, most genomes, 105 out of 134, clustered in a total of 21 clusters (Supplementary Fig. 2). The remaining 29 genomes did not show any significant relatedness to any other genomes. All showed >13 allele differences (Supplementary Fig. 2). Analysing WGS data for *P*. *larvae* can be a very useful tool to help identify connections between different beekeepers and beekeeping businesses to prevent further spread, and gain knowledge on how to prevent future outbreaks. The interpretation of the cgMLST results in this study is based on information from bee inspectors, advisors and beekeepers. More data on transfers, sales and other contacts between beekeeping operations need to be collected and compiled in order to make further conclusions. The majority of the 21 identified clusters could be linked to geographical areas (Fig. 4B). However, the largest cluster including 38 bacterial genomes from honeybee colonies in 10 counties could be associated with a big, beekeeping operation active in southern and central Sweden (Fig. 4C). Another smaller cluster including five bacterial isolates originating from three counties could be associated with a beekeeper selling and transferring beekeeping equipment and bees (Fig. 4D) (Supplementary Table 4).

## Discussion

Fast and accurate tools are necessary in disease outbreak situations, particularly for pathogens as deadly as *P*. *larvae*. Until recently, PCR‐based ERIC typing, Multiple Locus Variable number of tandem repeat Analysis and traditional MLST were the standard methods to identify genotypes within this species, allowing standardized typing capabilities and therefore allowing comparable results between laboratories (Alippi *et al*., 2004; Morrissey *et al*., 2015; Descamps *et al*., 2016). These methods, however, yielded relatively poor results for fine‐scale epidemiological studies.

In recent years, with the decrease in cost of WGS, modern typing methods include whole genomes and thousands of genes. The upscaling of subtyping by MLSTs offer an unprecedented discriminatory power that allows for, not only typing but also highly accurate outbreak investigations and disease tracing (Ghanem and El‐Gazzar, 2018; Mulhall *et al*., 2019; Sankarasubramanian *et al*., 2019).

In this study, we set out to build a standardized, stable cgMLST scheme that could be used across laboratories and genotypes in order to trace outbreaks with high resolution. A previous study has shown that cgMLST is a powerful tool for *P*. *larvae* epidemiological studies (Ågren *et al*., 2017). The 2017 study used two separate *ad hoc* cgMLST schemes for ERIC I and ERIC II, meaning the resolution for each ERIC type was very high. Here, using the same 24 samples belonging to an outbreak in 2014 in Gotland, Sweden, we have shown that our stable cgMLST scheme that includes both ERIC types shows comparable results and minimal loss of tracing power (Ågren *et al*., 2017).

Indeed, although the two ERIC type genomes differ significantly in size, with ERIC I having a 500 kb larger genome than ERIC II, the difference is mainly due to additional prophage sequences (Djukic *et al*., 2014) and has therefore little consequence on the development and effectiveness of the cgMLST scheme.

Once validated, we used the stable scheme to analyse 134 *P*. *larvae* genomes originating from symptomatic brood sampled in Sweden during 2016–2019. Using an allelic difference threshold of 10, we found clusters representing small and medium‐sized geographical areas, and also evidence of beekeeping transfers of the infection over larger geographical areas. An allelic threshold of 10 best highlighted the known differences between geographical locations and additional transmission information. This threshold is the same as the one identified by Ågren *et al*. (2017) during their outbreak tracing. It is important to note that establishing a threshold to detect significant differences between isolates can vary between studies and that this threshold should constitute more of a guideline than a fixed rule for future studies (Schurch *et al*., 2018).

Most of the identified clusters could be geographically linked and matched to information from bee inspectors and advisors. This is not surprising since most beekeepers in Sweden are hobbyists and keep their bees within limited geographical areas and robbing and drifting adult bees can spread the disease to neighbouring honeybee colonies and apiaries within their flying range. This illustrates the accuracy of the method but not least the added value of using the cgMLST scheme for disease tracing and epidemiological studies of *P*. *larvae*. More detailed information of transmission pathways will be of great value for the mitigation of future disease outbreaks, and the initial results will be subjected to further investigations and linked to supplemented data mining for more detailed information of the routes of AFB transmission in Sweden. Such results will serve as baseline data for legislation and prevention and as a basis for recommended improved management practices for affected beekeepers. Ultimately this will help ensure sustainable pollination services and global food security.

Although an SNP analysis could be used to slightly increase the resolution for outbreak tracing, a major drawback is that it is not standardized, needs a reference genome and requires time and expert analysis for every study (de Been *et al*., 2015; Ghanem and El‐Gazzar, 2018; Pearce *et al*., 2018; Schurch *et al*., 2018). This stable cgMLST can offer almost identical resolution and has the advantage of being a ‘ready to go’ solution, available for all laboratories to use. This tool will allow accurate tracing of *P*. *larvae* outbreaks in the future, therefore allowing better prevention of disease spread and destruction of important honeybee colonies.

## Experimental procedures

### Sequencing data

Paired‐end sequencing data were obtained from multiple sources (Supplementary Table 1). In the first part, 64 samples were sent to Anses from honeybee colonies with symptoms of AFB originating from 22 countries throughout Europe. Molecular diagnosis was confirmed by *16S rRNA* gene‐based PCR and ERIC typing was performed by rep‐PCR as previously described (Dobbelaere *et al*., 2001a; Genersch *et al*., 2006). DNA was extracted using the QIAmp® DNA Mini kit (Qiagen) and was quantified using Qubit™ dsDNA High Sensitivity Assay kit (Invitrogen™) following standard protocols. Whole‐genome paired‐end sequencing (2 × 100 nucleotides) was performed on an Illumina HiSeq 2500 instrument with the TruSeq Rapid kits (Illumina®). In the second part, 52 genomes were obtained from the Swedish National Veterinary Institute, SVA (ENA accession number PRJNA613377), methods described in Ågren *et al*. (2017) and 134 genomes of *P*. *larvae* from bacterial isolates originating from symptomatic brood sent to the Swedish National Reference Laboratory, SNRL, for Bee Health. The methods for bacterial isolation and DNA extraction followed published protocols (Nordström and Fries, 1995; Forsgren and Laugen, 2014). Additionally, 85 whole genome sequences from public databases were used (ENA accession number PRJEB1399). Finally, two complete genomes were included: NZ_CP019651 (ERIC I) as the seed genome and NZ_CP19652 (ERIC II) as an additional query sequence.

### Genome assembly

Raw paired‐end reads were quality assessed with FastQC and trimmed (ILLUMINACLIP:2:30:5:1:TRUE LEADING:3 TRAILING:3 SLIDINGWINDOW:4:20 MINLEN:36) with Trimmomatic 0.39 (Bolger *et al*., 2014). The Kraken software (Wood and Salzberg, 2014) was used to check reads for contamination. *De novo* assembly of reads was performed with SPAdes 3.14.1 (with the ‘‐careful’ setting) (Bankevich *et al*., 2012). The assemblies were error corrected using the Pilon software with default settings (Walker *et al*., 2014). Assembly quality was assessed by looking at contiguity (N50, number of contigs) and completeness (assembly size, GC%) (Supplementary Table 2). Samples that varied significantly in genome size (>5 Mb) or number of contigs (>2000) were discarded.

All raw reads generated were submitted to the European Nucleotide Archive (ENA) under the project numbers PRJEB40862 and PRJEB40534 for Anses and SNRL reads respectively.

### MLST sequence type

*Paenibacillus larvae* sequence types, STs, were identified using traditional MLST. Assembled genomes were queried using pubMLST.org, where strain information was given based on the seven‐loci scheme and ERIC type was subsequently inferred from the sequence type.

### cgMLST development

The genomes used to build the scheme were analysed using the core genome MLST (cgMLST) analysis software Ridom SeqSphere+ v7.1.0 (Ridom GmbH, Münster, Germany). The scheme was developed using NZ_CP019651 as seed genome and 53 query genome sequences that cover the genetic and geographical variability of the two ERIC types found in the dataset (Supplementary Table 1). Steps used to develop the standardized cgMLST schemes can be found in previous studies (Mellmann *et al*., 2011; Kohl *et al*., 2014; Antwerpen *et al*., 2015; Ghanem and El‐Gazzar, 2018). Briefly, the seed genome was selected using the following criteria: the assembly was complete, annotated and accessible, and the seed isolate was a common strain from the most predominant genotype found in infected colonies (Beims *et al*., 2020). The 53 query genomes were selected from an initial *ad hoc* cgMLST scheme where samples were selected to ensure all available clones were represented. For this, one or more representative genome(s) from each cluster, with 50 alleles or more difference from the closest neighbour in a minimum spanning tree, were selected (Ghanem *et al*., 2018). Plasmid genes were removed using plasmid assemblies available on NCBI (accession numbers: NZ_CP01953 and NC_023147.1).

Finally, the scheme was validated using an additional 148 genome assemblies spanning the whole available population genetic background of the species (Supplementary Table 1). If a scheme is stable, it is expected that each genome meets the 95% target match threshold as recommended in SeqSphere+ (Supplementary Table 3).

### cgMLST evaluation and outbreak analysis

Evaluation of this scheme was done in two parts. As a first step, 24 bacterial isolates from an isolated outbreak in 2014 on the island Gotland, Sweden, were analysed and compared with previously published molecular epidemiological results from the outbreak (Ågren *et al*., 2017). This dataset was explored as the previous study used two *ad hoc* schemes, one for each ERIC type individually. If the stable scheme is to add value to the scientific community, it needs to be easy to use (one scheme for both genotypes) and also provide high‐resolution typing for closely related samples.

Second, the scheme was used to evaluate 134 bacterial strains isolated from symptomatic brood from 10 counties in South and Central Sweden. The samples were loaded into SeqSphere+ and typed using the 2419 cgMLST gene targets.

## Supporting information


**Supplementary Figure 1.** Histograms showing cgMLST loci count against the number of allelic variation per locus. A) Count of cgMLST loci for different allele counts (1–26). This figure shows that the majority of loci have low allelic variation B) Count of isolates with a missing allele in each different loci for different allele counts (1–26). For example, across loci that have 5 different alleles, a total of 70 isolates have missing alleles in those loci. This figure shows that there are more missing targets in loci with low allelic variation. This is not surprising, as low variation loci are more numerous than high variation loci. The high count of isolates with missing targets in loci with low variability are mainly due to loci missing in isolates of ERIC II genotypes. This was expected due to the large genomic differences between ERIC I and ERIC II genotypes. Although, as shown in this study, these had little impact on the resolution of the scheme. There is a high peak of missing targets in loci with 14 alleles in B). This is due mainly to one locus, ERICI_RS19850, which is absent in 28 isolates all belonging to the sequence types ST19 and ST5. This would warrant further investigation in the genomic differences of these strains. Loci with high allelic variation are usually discarded from cgMLST schemes as they can be unstable and not present in all isolates. However, for this scheme, as B) shows, those loci were conserved as they were present in the wide variety of isolates in this study.

**Supplementary Figure 2.** Cluster analysis of the 134 SNRL isolates based on allelic differences using the stable core genome MLST. A minimum spanning tree showing number of allelic differences between isolates. Results are based on 2419 target genes. Identified clusters of <10 allelic differences are in coloured groups with grey background showing their links. All isolates in white are >10 allelic differences from their closest neighbour and therefore were not grouped in any particular cluster.

**Supplementary Table 1.** Details of samples used in study. Includes sample name, sample origin, year of sampling, location, latitude and longitude coordinates where relevant, the sequencing platform used, ERIC type, strain identified by MLST, number of contigs, number of bases, longest contig, N50 and GC content
**Supplementary Table 2.** 7‐gene MLST allelic profile for eight samples that could not have an ST attributed to them using the traditional typing scheme. NA denotes the loci that were not found in each sample.
**Supplementary Table 3.** cgMLST scheme. This data is available on https://www.cgmlst.org/ncs
**Supplementary Table 4.** Details of SNRL samples used in study. Includes sample name, year of sampling, location, latitude and longitude coordinates, cluster ID number and the type of connection of isolates in a cluster.
