# Supplementary figures and images for: Development and evaluation of a core genome multilocus sequence typing scheme for Paenibacillus larvae, the deadly American foulbrood pathogen of honeybees
Source: Environ Microbiol. 2021 Mar 2;23(9):5042–51. doi: 10.1111/1462-2920.15442 (PMC8518682; doi:10.1111/1462-2920.15442)

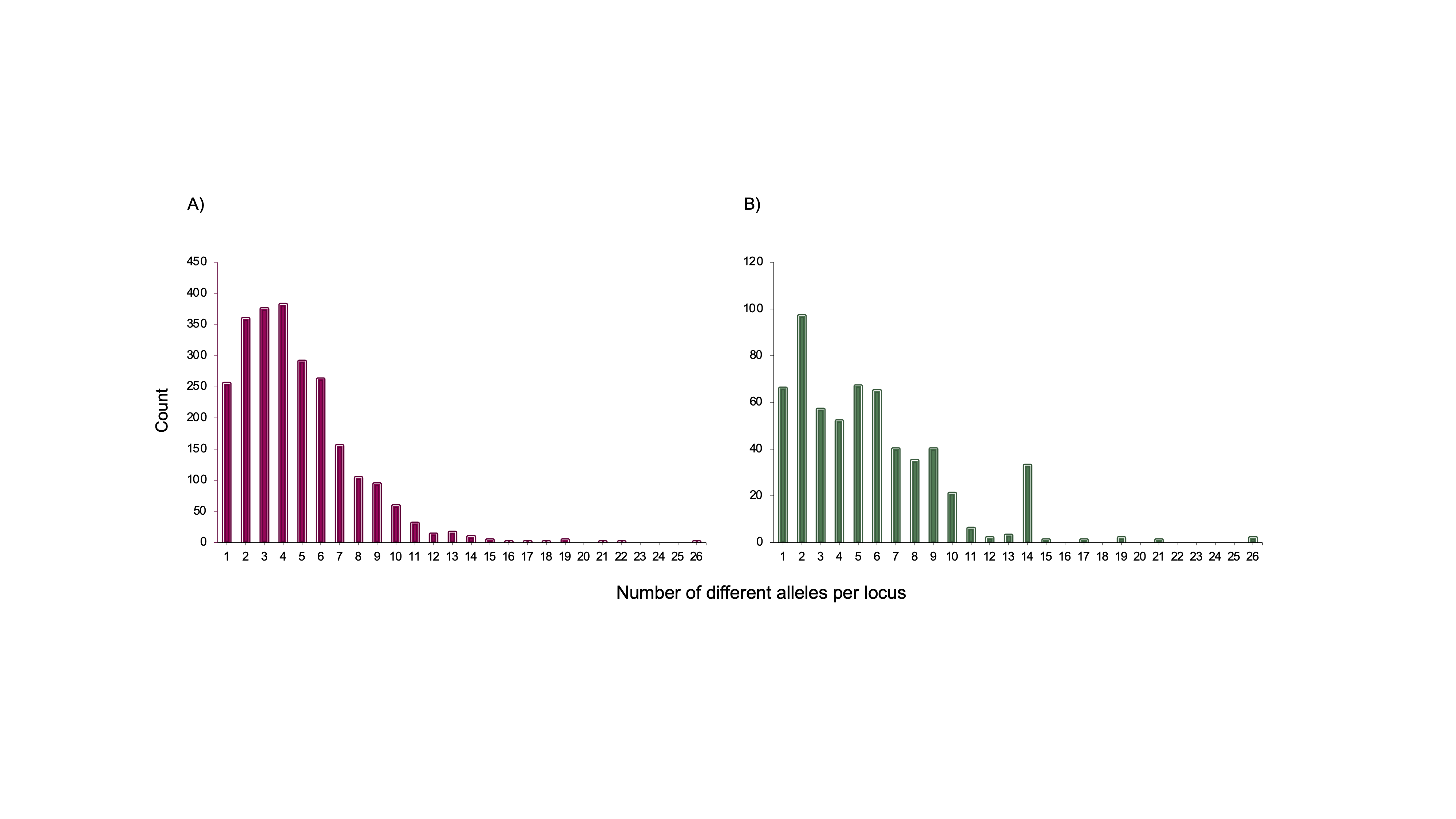

Supplement: Supplementary file 1 — Supplementary Figure 1. Histograms showing cgMLST loci count against the number of allelic variation per locus. A) Count of cgMLST loci for different allele counts (1–26). This figure shows that the majority of loci have low allelic variation B) Count of isolates with a missing allele in each different loci for different allele counts (1–26). For example, across loci that have 5 different alleles, a total of 70 isolates have missing alleles in those loci. This figure shows that there are more missing targets in loci with low allelic variation. This is not surprising, as low variation loci are more numerous than high variation loci. The high count of isolates with missing targets in loci with low variability are mainly due to loci missing in isolates of ERIC II genotypes. This was expected due to the large genomic differences between ERIC I and ERIC II genotypes. Although, as shown in this study, these had little impact on the resolution of the scheme. There is a high peak of missing targets in loci with 14 alleles in B). This is due mainly to one locus, ERICI_RS19850, which is absent in 28 isolates all belonging to the sequence types ST19 and ST5. This would warrant further investigation in the genomic differences of these strains. Loci with high allelic variation are usually discarded from cgMLST schemes as they can be unstable and not present in all isolates. However, for this scheme, as B) shows, those loci were conserved as they were present in the wide variety of isolates in this study. [file EMI-23-5042-s001.tif]

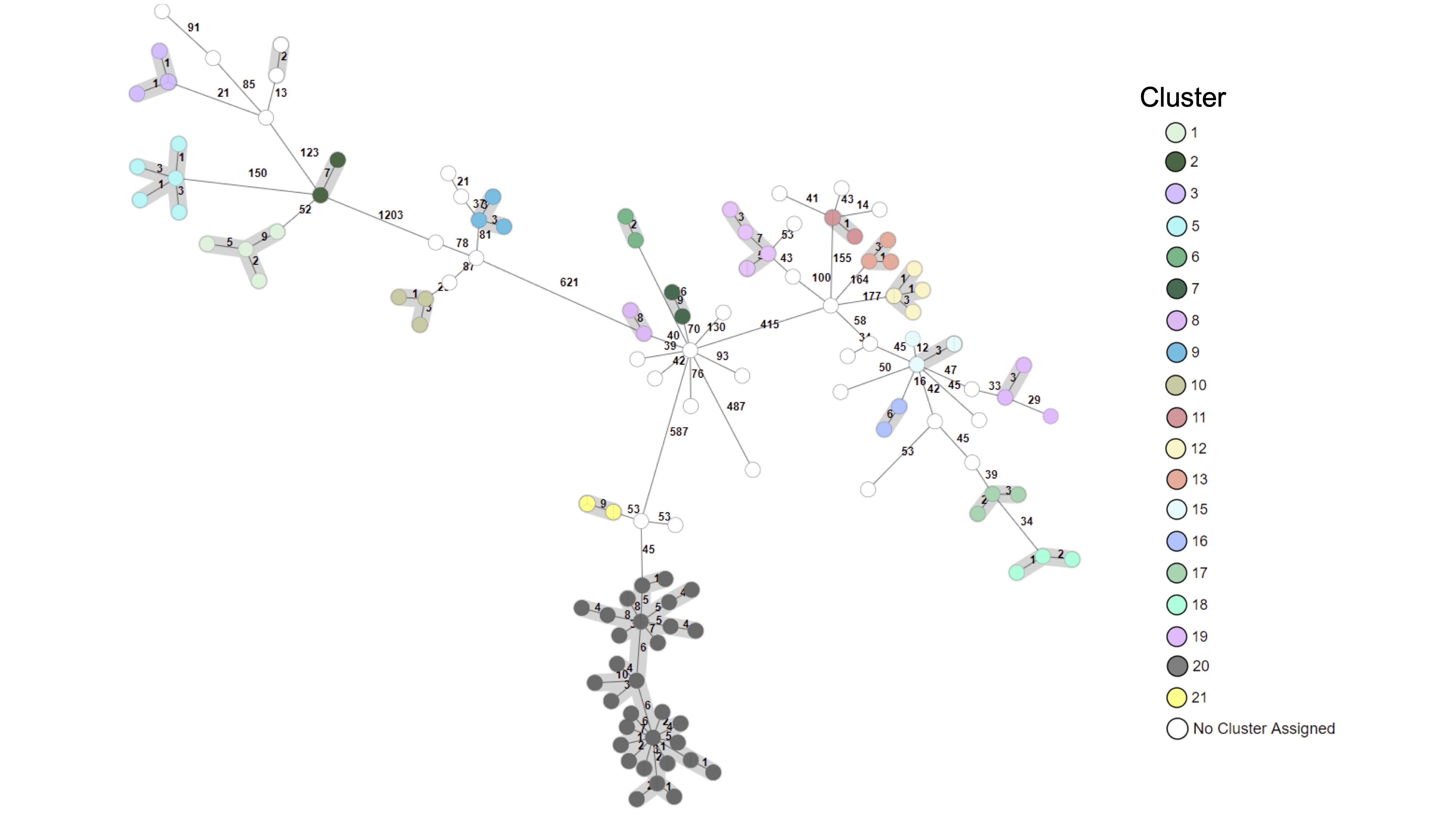

Supplement: Supplementary file 2 — Supplementary Figure 2. Cluster analysis of the 134 SNRL isolates based on allelic differences using the stable core genome MLST. A minimum spanning tree showing number of allelic differences between isolates. Results are based on 2419 target genes. Identified clusters of <10 allelic differences are in coloured groups with grey background showing their links. All isolates in white are >10 allelic differences from their closest neighbour and therefore were not grouped in any particular cluster. [file EMI-23-5042-s002.tif]
